# The immune landscape in BCR‐ABL negative myeloproliferative neoplasms: inflammation, infections and opportunities for immunotherapy

**DOI:** 10.1111/bjh.17850

**Published:** 2021-10-07

**Authors:** Marie Strickland, Lynn Quek, Bethan Psaila

**Affiliations:** ^1^ MRC Molecular Haematology Unit MRC Weatherall Institute of Molecular Medicine, University of Oxford Oxford; ^2^ National Institutes for Health Research Biomedical Research Centre University of Oxford Oxford; ^3^ Department of Haematological Medicine King's College Hospital NHS Foundation Trust London; ^4^ Department of Haematology, School of Cancer and Pharmaceutical Sciences King's College London London UK

**Keywords:** Chronic myeloid malignancies, immunity, immunocompromise, immuno‐oncology, JAK inhibitors

## Abstract

Breakpoint cluster region‐Abelson (BCR‐ABL) negative myeloproliferative neoplasms (MPNs) are chronic myeloid neoplasms initiated by the acquisition of gene mutation(s) in a haematopoietic stem cell, leading to clonal expansion and over‐production of blood cells and their progenitors. MPNs encompass a spectrum of disorders with overlapping but distinct molecular, laboratory and clinical features. This includes polycythaemia vera, essential thrombocythaemia and myelofibrosis. Dysregulation of the immune system is key to the pathology of MPNs, supporting clonal evolution, mediating symptoms and resulting in varying degrees of immunocompromise. Targeting immune dysfunction is an important treatment strategy. In the present review, we focus on the immune landscape in patients with MPNs – the role of inflammation in disease pathogenesis, susceptibility to infection and emerging strategies for therapeutic immune modulation. Further detailed work is required to delineate immune perturbation more precisely in MPNs to determine how and why vulnerability to infection differs between clinical subtypes and to better understand how inflammation results in a competitive advantage for the MPN clone. These studies may help shed light on new designs for disease‐modifying therapies.

## The role of innate and adaptive immunity in myeloproliferative neoplasms (MPNs)

The innate and adaptive immune systems work synergistically to protect against the emergence of cancer via a series of stepwise events referred to as the ‘cancer‐immunity cycle’.^1^ This process depends on the release of aberrant proteins by malignant cells and their detection and capture by antigen‐presenting cells (APCs) of the innate immune system (e.g. dendritic cells and macrophages). APCs then prime and activate effector T cells, leading to target elimination. This process is carefully moderated by co‐stimulatory and co‐inhibitory signals and the ratio of effector *versus* regulatory T cells (Tregs). Cancer cells must evade this cycle in order to survive and proliferate. This is achieved via mechanisms that downregulate anti‐tumoral immunity, such as reduced expression of human leucocyte antigen (HLA) class I molecules preventing antigen presentation, or upregulation of molecules such as programmed death‐ligand 1 (PD‐L1) that inhibit T‐cell activity (reviewed in Sukari *et al*.^2^ and Sánchez‐Paulete *et al*.^3^).

In the present review, we first discuss data indicating how MPN driver mutations result in inflammatory signalling, and how this is permissive for clonal expansion and disease progression. The mechanisms by which the MPN clone evades anti‐tumoral immunity and the impact of immune perturbations on susceptibility to infection are then considered. Finally, emerging strategies for targeted immunomodulatory therapy for MPNs are discussed.

## MPN gene mutations result in increased production of inflammatory cytokines from both clonal and non‐clonal cells

Myeloproliferative neoplasms arise following the acquisition of gene mutations in haematopoietic stem cells (HSCs) that result in cytokine‐independent activation of Janus kinase‐signal transducer and activator of transcription (JAK‐STAT) signalling. The most common mutations occur in the genes encoding *JAK2* (*JAK2* ^V617F^), calreticulin (*CALR)* and the thrombopoietin receptor, *MPL*. MPN ‘driver’ mutations result in the activation of pro‐inflammatory signalling, in particular tumour necrosis factor (TNF)/nuclear factor κ‐light‐chain‐enhancer of activated B cells (NF‐κB) pathways, in mutated HSCs and their progeny.^4^, ^5^, ^6^ Studies at the single cell level have shown that the increased production of inflammatory cytokines results from both an increase in the percentage of cytokine‐secreting cells, as well as augmented cytokine secretion *per cell*.^4^ In addition, in patients with mutant (mut)*CALR*‐driven essential thrombocythaemia (ET) and myelofibrosis, there is a direct pro‐inflammatory effect of the mutant protein itself, as secreted mutCALR exaggerates cytokine production from normal monocytes.^7^

**Fig 1:**
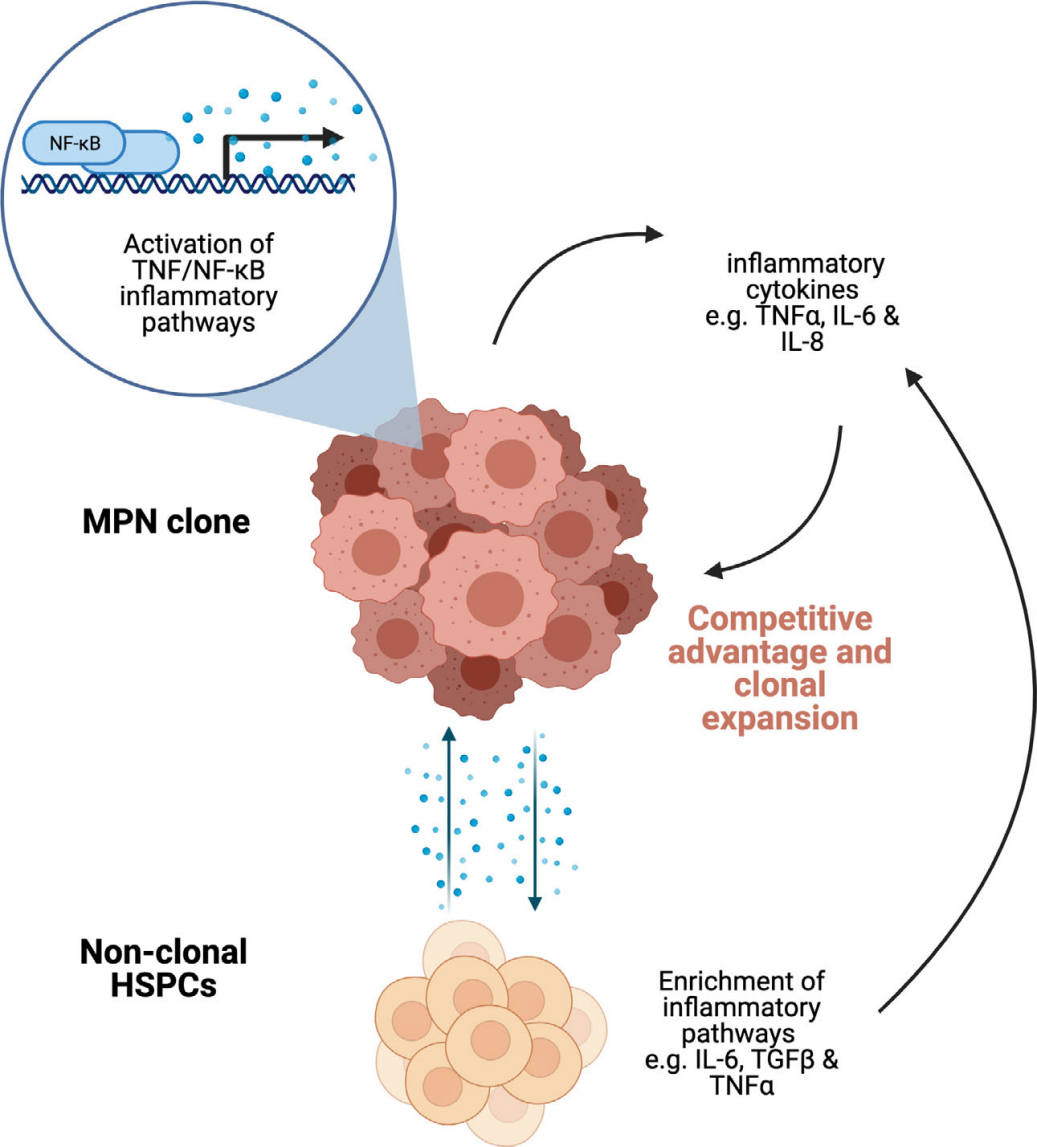
Pro‐inflammatory interactions between clonal and non‐clonal cells. Inflammatory cytokines and growth factors such as TNFα, IL‐6, and IL‐8 confer a competitive advantage to the malignant MPN clone and are associated with increased symptom burden. *JAK2* ^V617F^ mutations also alter the epigenetic regulation of TNF/NF‐κB inflammatory signalling pathways, further increasing inflammation. Extra‐cellular signals released by the MPN clone result in excess production of inflammatory cytokines by non‐clonal myeloid cells, lymphoid cells and bone marrow stroma. HSPCs, haematopoietic stem and progenitor cells; IL, interleukin; JAK2, Janus kinase 2; MPN, myeloproliferative neoplasm; NF‐κB, nuclear factor κ‐light‐chain‐enhancer of activated B cells; TGFβ, transforming growth factor beta; TNF, tumour necrosis factor.

In addition to production of inflammatory cytokines by the MPN clone, immune dysregulation also results from paracrine/endocrine effects on non‐clonal haematopoietic and stromal cells (Fig 1). For example, in patients with myelofibrosis, Wang *et al*.^8^ observed that excessive soluble interleukin‐2 receptor α (IL‐2Rα) was produced by non‐clonal cluster of differentiation (CD)4^+^CD25^+^ forkhead box protein 3 (FoxP3)^+^ Tregs. This has also been carefully documented by studies at the single cell level in which accurate discrimination of mutated and wild‐type cells can be performed in parallel with transcriptomic analysis. For example, simultaneous interrogation of clonal and non‐clonal populations in individuals with BCR‐ABL‐negative MPNs, as well as chronic myeloid leukaemia (CML), showed significant enrichment of inflammatory pathways including IL‐6, transforming growth factor beta (TGFβ) and TNFα‐associated signalling in wild‐type HSCs in patients with CML^9^ and in patients with *JAK2* ^V617F+^ myelofibrosis,^10^ in comparison to HSCs in age‐matched, healthy donors. In pre‐clinical models, mice injected with *JAK* ^V617F+^ cells demonstrated increased TNFα and IL‐6 production by host cells in response to proteins secreted by the malignant clone.^11^

## Inflammation is permissive for clonal expansion and MPN disease progression

Recent data suggest that MPN mutations may be acquired *in utero* or during early childhood, although they typically do not manifest until adult life.^12^, ^13^ This suggests a model in which progression to clinically overt disease may be driven by gradual clonal expansion supported by ageing‐associated immune dysfunction and inflammatory bone marrow changes.^14^, ^15^, ^16^ In support of this hypothesis, certain inflammatory cytokines that are increased in patients with MPN, including TNFα and interferon α (IFNα), have been shown to confer a selective growth advantage to *JAK2* ^V617F^‐mutant over wild‐type cells *in vitro*,^17^, ^18^ enabling clonal expansion (Fig 1). Recently published data indicates that JAK2V617F‐mutant HSCs are hyper‐responsive to IFN, and that IFN signalling promotes megakaryocyte‐biased haematopoiesis, which may be an important determinant of MPN phenotype and disease evolution.^19^, ^20^ Increased TNFα as well as hepatocyte growth factor (HGF), platelet‐derived growth factor (PDGF), vascular endothelial growth factor (VEGF), and interleukins especially IL‐6 and IL‐8 have also been associated with increased *JAK2* ^V617F^ mutation burden and poor outcomes.^17^, ^21^, ^22^

Associations have also been noted between certain cytokines and disease stage and/or fibrotic progression. In a study of >120 patients with myelofibrosis, IL‐8 and the IL‐2R were highly predictive of disease stage, with increased levels of one or both associated with an increased Dynamic International Prognostic Scoring System (DIPSS) risk score.^22^ Higher levels of soluble IL‐2Rα (sIL‐2Rα) have also been observed in patients with myelofibrosis relative to either patients with polycythaemia vera (PV)/ET or healthy controls^8^ and increased IL‐6, IL‐2 and sIL‐2Rα have been demonstrated in patients following progression from ET and PV to myelofibrosis.^23^ Further support for a key role of inflammation in MPN progression is that the development of fibrosis in myelofibrosis has been reported in some studies to correlate more strongly with the levels of certain cytokines including IL‐8 and TGF‐β than malignant clone burden (e.g. *JAK2* ^V617F^ variant allele frequency).^24^, ^25^ In addition to supporting clonal expansion, inflammatory cytokines result in high symptom burden for some patients, including fatigue, itching, sweats and weight loss.^26^

## Breaking of the tumour‐immunity cycle and evasion of anti‐tumour immunosurveillance

Subversion of innate and adaptive immune surveillance and breaking of the tumour‐immunity cycle contribute to enabling the neoplastic myeloid clone to expand. APCs in patients with MPNs have a lower ability to process and present antigens, leading to suboptimal priming and activation of T cells. Both class I and II *HLA* genes are down‐regulated in PV, ET and myelofibrosis, with progressive downregulation of certain genes [*BAT2L*, HLA Complex Group 11 (*HCG11*) and major histocompatibility complex (MHC), Class I‐related (*MR1*)] in ET *versus* PV *versus* myelofibrosis.^27^ Numbers of circulating dendritic cells are reduced in patients with myelofibrosis, most significantly in those with the *JAK2* ^V617F^ mutation.^28^ Alongside this, the differentiation of monocytes into dendritic cells *in vitro* has been shown to be impaired in a study of patients with myelofibrosis, with an associated defect in their capacity to prime T cells.^28^ An increase in myeloid‐derived suppressor cells (MDSCs) has also been documented, specifically in myelofibrosis.^29^ MDSCs lack expression of the MHC class II receptor HLA‐DR and have an overall immunosuppressive impact on T lymphocytes, aiding immune evasion. Loss or down‐regulation of HLA class I antigens results in escape from immune surveillance in several solid tumours and other haematological malignancies.^30^

In addition to impaired antigen presentation and T‐cell priming, mutations in *CALR* also subvert cellular immunity via a specific mechanism, as the normal CALR protein has a key role in tumour immunosurveillance. CALR expressed on the cell surface promotes phagocytosis of malignant cells by engaging low‐density lipoprotein receptor‐related protein (LRP) on macrophages.^31^ To evade phagocytosis, neoplastic cells upregulate expression of CD47, a cell surface molecule that binds signal‐regulatory protein α (SIRP‐α) on macrophages and delivers a ‘do not eat me’ signal.^32^ An altered ratio of expression of pro‐phagocytic CALR and anti‐phagocytic CD47 mediates evasion from immunosurveillance and is the basis for CD47‐targeting molecules as novel anti‐cancer therapies.^33^ In patients with mut*CALR^+^* MPN, mutant CALR protein inhibits phagocytosis of apoptotic MPN cells by dendritic cells, preventing effective antigen presentation.^34^

Alterations in immune effector cell number and function have been reported, including a reduced number of CD56^+^ CD3^−^ natural killer (NK) cells in untreated patients with MPN compared to healthy controls,^35^ a reduced number of CD3^+^ T cells,^36^ and impaired IFNγ production by T cells.^22^ Unlike in solid cancers where Tregs are generally increased, numbers of Tregs do not appear to be substantially increased in MPNs.^8^, ^36^ However, their ability to produce soluble IL‐2Rα is reduced in myelofibrosis relative to other MPN diagnoses and compared to healthy controls.^8^

Myelofibrosis in particular is associated with pronounced changes to the bone marrow stroma and non‐cellular matrix. It is possible that these structural changes may prevent immune cell surveillance within the bone marrow microenvironment, coupled with immune ‘exhaustion’ induced by chronic and high‐level exposure to the MPN cancer antigens.^37^, ^38^ Together, these changes impact the efficiency of identification and targeting of the MPN clone by innate and adaptive immune mechanisms.

## Disease‐mediated susceptibility to infection

The altered immune landscape in MPNs results in an increased incidence of bacterial, viral, and fungal infections. A recent population‐based cohort study including >8000 patients with MPNs and 32 000 controls in Sweden identified a hazard ratio (HR) of ~2·0 for both bacterial and viral infections across all MPN subgroups.^39^ The highest risk of any infection was in those with myelofibrosis (HR 3·7) compared to those with PV or ET (HR 1·7), and there was no difference in infection rates in patients with ET or PV who were not receiving treatment compared to those who were receiving hydroxyurea, IFN or anagrelide.^39^ This suggests that a large component of susceptibility to infection is mediated by the underlying disease and present to varying degrees in all three MPN subtypes, irrespective of immunocompromise resulting from particular treatments. As for many other haematological disorders, infection rates are particularly high in those who are transfusion dependent with iron overload.^40^

Vulnerability to infection in patients with MPN was rapidly highlighted by the coronavirus disease 2019 (COVID‐19) pandemic. Several studies documented a substantially higher risk of severe infection and death from COVID‐19 in patients with haematological malignancy, including those with MPNs.^41^, ^42^, ^43^, ^44^ In a systematic review and meta‐analysis of 3377 patients with haematological malignancies and COVID‐19, risk of death in patients with MPNs was 34%, substantially higher than in the general population, although lower than for those with acquired bone marrow failure syndromes and acute leukaemias.^45^ A UK‐wide national audit of COVID‐19 in MPN highlighted the strong correlation between advanced age and male sex with severe COVID‐19 within MPN patients, similar to that observed in the general population.^46^ In this study, patients not receiving cytoreductive therapy were under‐represented in the cohort, suggesting a lower risk in untreated patients, while ruxolitinib‐treated patients were over‐represented, although this study was not able to assess the relative impact of a more advanced underlying disease or age and comorbidities *versus* a direct association with ruxolitinib treatment itself. A particularly poor outcome was observed in ruxolitinib‐treated patients aged >75 years.^46^ One USA study suggested a substantially stronger effect of recently diagnosed ET on the odds of COVID‐19 infection as compared to PV.^43^

As a result of increased susceptibility to infection in MPN and higher risk of developing serious complications, most guidelines recommend that patients with MPN receive the annual influenza vaccine and other inactivated vaccines but that live attenuated vaccines (e.g. shingles vaccine) are avoided. However, responses to vaccination in some patients with MPN may be suboptimal, with evidence, albeit in a small cohort, of delayed and impaired B cell and T cell memory responses 3‐weeks and 3‐months after Influenza A vaccination.^47^ Evidence of significantly reduced seroconversion rates and lower antibody titres has also been documented in patients with MPNs after the first dose of the COVID‐19 vaccine, particularly in patients with ET and PV receiving hydroxycarbamide and patients receiving ruxolitinib, while responses in patients with ET and PV treated with pegylated IFN were relatively unimpaired.^48^

## Immunomodulatory impact of current cytoreductive MPN treatments

Susceptibility to infection is a well‐documented consequence of ruxolitinib, a JAK1/JAK2 inhibitor that is highly effective at reducing symptoms and splenomegaly patients with symptomatic myelofibrosis. A recent study of 948 patients with MPN in Germany and Italy found that the proportion of patients experiencing infections was significantly higher in patients receiving ruxolitinib or ruxolitinib combinations as compared to patients not receiving any treatment or hydroxycarbamide alone, and that ruxolitinib‐treated patients were more likely to require hospital outpatients visits or admission for infections.^49^ This finding was observed for both upper respiratory tract and gastro‐intestinal infections.^49^ Of note, there was no difference in infection rates between patients with different driver mutation status (*JAK2* ^V617F^, *CALR*, *MPL*, triple negative) but patients with myelofibrosis had significantly more infections than those with ET or PV.^49^ One consideration in interpreting these data is that generally patients treated with ruxolitinib tend to be older and with more advanced disease, and non‐drug related factors may contribute to their vulnerability to infection. Certain specific infections such as herpes virus reactivation and urinary tract infections are more prevalent in patients with MPN treated with ruxolitinib,^50^ and reactivation of rare opportunistic infections have also been reported, including progressive multifocal leukoencephalopathy (PML),^51^ hepatitis B,^52^ pneumonia and disseminated tuberculosis.^53^, ^54^, ^55^, ^56^ As a result, patients should be screened for latent and atypical infections such as hepatitis C and B and tuberculous, and prophylactic acyclovir considered to prevent shingles, prior to the commencement of ruxolitinib treatment.

The mechanism of ruxolitinib‐induced immunosuppression is multifactorial. The success of ruxolitinib in reducing symptoms and spleen sizes is largely due to its strong anti‐proliferative and anti‐inflammatory properties, with clear evidence of a reduction in circulating inflammatory cytokines including TNFα, IL‐1, IL‐6, IL‐8, IL‐1RA and IFNγ and reduced cytokine‐mediated symptoms.^57^, ^58^ Ruxolitinib treatment results in impaired effector T‐cell function,^59^ as well as changes in Tregs, NK and dendritic cells,^60^, ^61^, ^62^ reviewed in McLornan *et al*.^63^ As the effects on T cells are largely due to the inhibition of JAK1, it is likely that JAK inhibitors that are more selective for JAK2, such as fedratinib and pacritinib, may be associated with a lower risk of infection.^64^ However, whilst the trial data are promising, more time is needed to properly evaluate the impact of these ‘second‐generation’ JAK inhibitors on susceptibility to infection.

There does not appear to be a substantial increase in infections in patients with MPN receiving IFNα or hydroxycarbamide treatment as compared to patients not receiving cytoreductive treatment.^49^ IFNα has been used for many decades as an immunomodulatory agent in MPN and remains the only licenced agent for which complete or major molecular remissions have been observed, supporting the hypothesis that modulating the immune system is important for clonal response.^18^ Patients treated with IFNα have an altered balance of regulatory *versus* effector T cells, with studies reporting double the proportion of CD25^+^FoxP3^+^ Tregs in the CD4^+^ T‐cell compartment as compared to untreated patients or healthy donors,^65^ and increased numbers of effector T cells.^66^ However, it is unclear whether or how this mediates clonal responses.

## Novel strategies for targeted immunotherapy in MPN

Many modern immunotherapy strategies have been pioneered in haematological cancers, such as immune checkpoint blockade in lymphomas, chimeric antigen receptor (CAR)‐T cell therapies in B‐cell neoplasms and antibody therapies in acute myeloid leukaemia (AML). Obstacles to similar successes in MPNs include a lack of ubiquitous and MPN clone‐specific cell surface targets, substantial cellular and/or molecular heterogeneity between patients and between disease subtypes, and an inability of the therapies to ignite anti‐tumour immunity due to the immune derangement resulting from the myeloid neoplasm.

**Fig 2:**
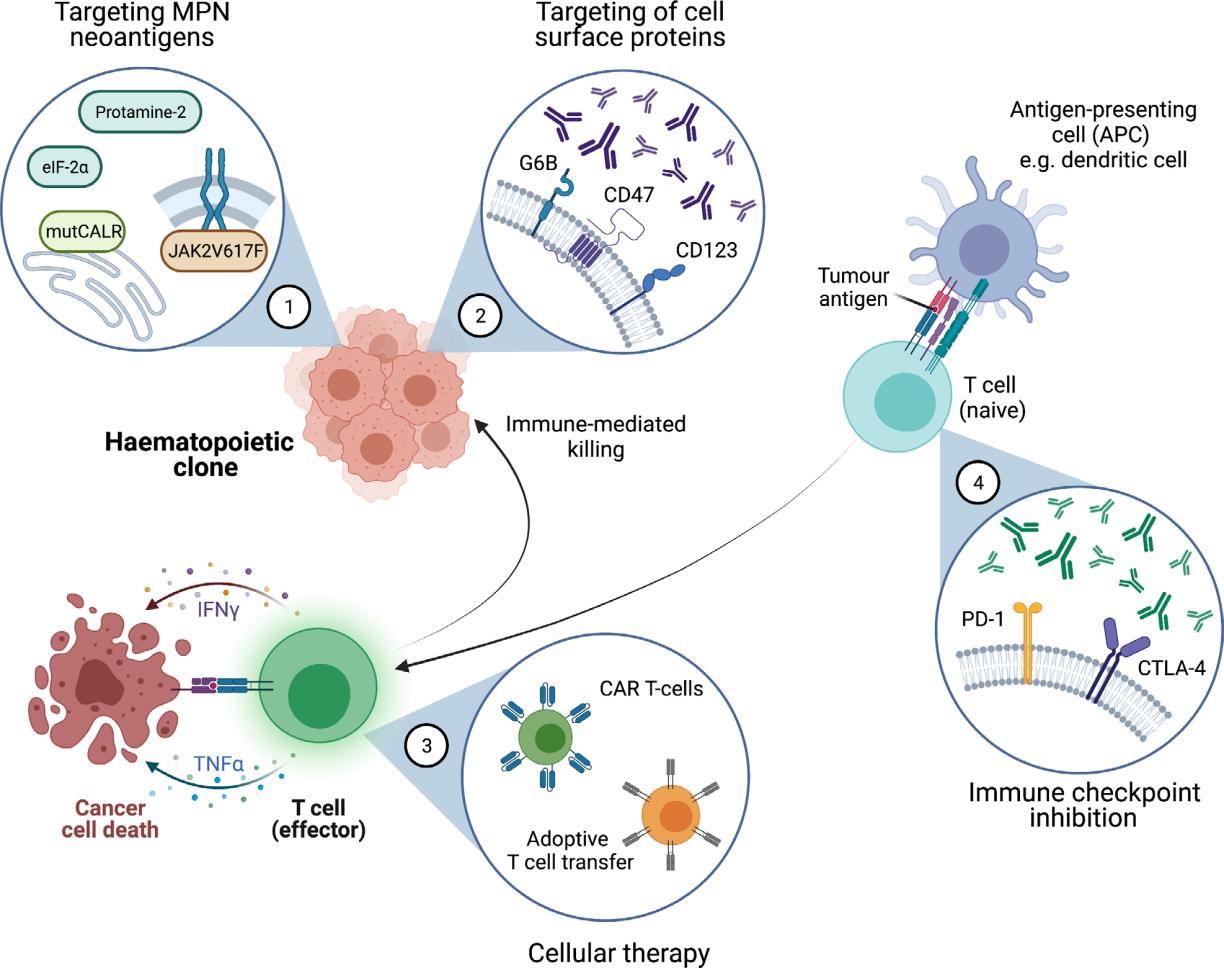
Opportunities for immune‐based therapies in myeloproliferative neoplasm (MPN). (1) Identification of MPN neoantigens that elicit immune responses, such as *JAK2* ^V617F^ and mutant *CALR* epitopes, and alternatively spliced proteins, such as eIF‐2α and protamine‐2 could be exploited for immune therapy; (2) Antibody therapies can be directed against cell surface proteins aberrantly expressed on the cell surface of MPN clone cells; (3) CAR‐T cells and adoptive T‐cell transfer confer T cells with the ability to target a specific protein, enhancing T‐cell recognition and destruction of mutant cells; (4) Blockade of the PD‐1 and CTLA‐4 inhibitory pathways on T cells enables better activation of naïve T cells by APCs such as dendritic cells, which present tumour antigens to the T cells for recognition. APC, antigen‐presenting cell; CALR, calreticulin; CAR, chimeric antigen receptor; CD, cluster of differentiation; CTLA‐4, cytotoxic T‐lymphocyte protein 4; eIF‐2α, eukaryotic initiation factor‐2α; IFN, interferon; JAK2, Janus kinase 2; MPN, myeloproliferative neoplasm; NF‐κB, nuclear factor κ‐light‐chain‐enhancer of activated B cells; PD‐1, programmed cell death‐protein 1; TNF, tumour necrosis factor.

There are many new therapies in clinical development for MPNs, including novel JAK inhibitors that are more selective for JAK2, ‘add‐on’ drugs that improve efficacy of ruxolitinib both in ruxolitinib‐naïve patients and in those who have a suboptimal response, and new targets (Table I and reviewed in Venugopal and Mascarenhas).^67^ Many of these have anti‐inflammatory activity, such as the bromodomain and extra‐terminal motif (BET)‐inhibitor CPI‐0610·^68^ In the next section, we outline some emerging targeted immunotherapy strategies that aim to ameliorate the deficiencies in anti‐tumoral immunity described above (Fig 2; also reviewed in Holmstrom *et al*.^69^).

**Table I:** Examples of immune modulating therapies employed in myeloproliferative neoplasms.

	Strategy	Disease	References
Immunomodulatory agents currently in use	Interferon‐α	Myelofibrosis, PV and ET	91, 92, 93
	Haematopoietic stem cell transplantation	Myelofibrosis	94, 95
	Corticosteroids	Myelofibrosis	96
	JAK inhibitors – ruxolitinib, fedratinib, pacritinib, momelotinib	Myelofibrosis, PV and ET	97, 98, 99, 100
	Immunomodulators – thalidomide, lenalidomide, pomalidomide	Myelofibrosis	101, 102, 103
Targeted immunomodulatory agents in clinical‐stage development	Anti‐CD123 – tagraxofusp	Myelofibrosis	104
	mutCALR vaccination	Myelofibrosis and ET	88
	Anti‐PD‐1 – pembrolizumab, nivolumab	Myelofibrosis	89, 105

CD, cluster of differentiation; ET, essential thrombocythaemia; JAK, Janus kinase, mutCALR, mutant calreticulin; PD‐1, programmed cell death‐protein 1; PV, polycythaemia vera.

## Identifying neoantigens in MPNs

Tumour neoantigens are cancer cell‐specific molecules that activate T cells through MHC class I and II presentation by APCs. Neoantigens can result from gene mutations, fusions, alternative splicing and post‐translational protein modifications. Selective CD8^+^ cytotoxic T‐cell reactivity against epitopes within *JAK2* ^V617F^ have been documented.^70^ In this study, dendritic cell‐mediated stimulation of CD8^+^ T cells induced a significant release of IFNγ and TNFα, two markers of T‐cell activation, although this release required co‐stimulation with IL‐2, IL‐7 and IL‐12.^70^ In contrast to the *JAK2* ^V617F^ mutation, which is a single amino acid change, mutant CALR protein represents a large neoantigen that is expressed on the cell surface, and therefore is an attractive target for tumour‐specific immunotherapy in patients with *mutCALR*‐driven MPN. Immunoreactivity to mutant CALR has been observed in a subset of patients with MPN.^36^, ^71^ Responses against two neoantigens, CALRlong1 and CALRlong2 were reported in 50% and 42% of patients respectively. The response rate was found to be higher in ET than for myelofibrosis, with 80% of the patients with ET studied showing a response compared to only 36% of the patients with myelofibrosis.^71^ Notably, memory T‐cell responses to CALR mutant peptides have also been detected in healthy donors without MPN.^36^ This suggests that CALR may be a mutation that is frequently generated but held in check by the immune system, and that an inability to invoke responses against CALR mutant peptides may mediate immune evasion in patients who develop overt disease.

In MPNs, tumour neoantigens have also been identified by screening expression complementary DNA (cDNA) libraries with sera from patients. This method identified that peptides normally expressed only in the human testis, an immune‐privileged site, are over‐expressed in both CML and PV.^72^, ^73^ In one study, two proteins, eukaryotic translation initiation factor‐2A (eIF‐2A) and protamine 2, were shown to elicit immunoglobulin G (IgG) antibody reactions, in particular in those patients treated with IFNα, although expression was not specific to the mutant clone and there was no difference in expression levels of these antigens between clonal and wild‐type cells.^73^

A recent study examining the granulocyte transcriptome profile in >100 patients with MPNs using RNA‐sequencing identified 13 gene fusions, 231 non‐synonymous single nucleotide variants and 21 insertions and deletions in 106 of 113 patients.^74^ Using a ‘virtual peptide library’, the authors predicted that both *CALR* and *JAK2* mutations resulted in a large number of neoantigens and 35 of these peptides were predicted to be strong MHC class I binders. The frequency of neoantigens was higher in patients with splicing factor 3B subunit 1 (*SF3B1*) mutations. This suggests a role for transcriptomics in designing personalised vaccine or adoptive cell‐based therapies.^74^

## Targeting the MPN clone with therapeutic antibodies

Antibody‐based therapies may be used to deliver cytotoxic drugs (e.g. antibody drug conjugates), induce antibody‐dependent cellular cytotoxicity (ADCC), or augment complement‐mediated cytotoxicity or the innate immune response. CD123, the receptor for IL‐3, has been identified as a therapeutic target in several myeloid malignancies including MPNs. Tagraxofusp is a targeted therapy directed to CD123 that comprises recombinant IL‐3 fused to a truncated diphtheria toxin. Following United States Food and Drug Administration (FDA) approval in blastic plasmacytoid dendritic neoplasm, a Phase I/II trial of tagraxofusp in MPN demonstrated some clinical efficacy, highlighting a potential role for CD123 targeting in this setting.^75^

Single cell technologies are also increasingly being employed to uncover cell type‐specific targets. ‘Multi‐omic’ methods that enable simultaneous detection of gene mutations, transcriptome analysis and immunophenotyping of individual cells^10^, ^76^, ^77^, ^78^ offer the necessary resolution to identify cancer cell‐specific targets. For example, expression of the megakaryocyte gene *G6B* was found to be dramatically increased in stem/progenitor cells in myelofibrosis, specifically in *JAK2* ^V617F^‐mutant *versus* wild‐type stem cells within the same patients.^79^ G6B was validated *in vitro* as a potential immunotherapy target, capable of supporting receptor‐mediated internalisation of a tool antibody.

As discussed previously, CD47 represents an attractive target for activating the innate immune system. Targeting CD47 blocks the ‘do not eat me’ signal from leukaemic cells resulting in their elimination by macrophages.^80^ The use of targeting antibodies against CD47 are currently under development for the treatment of AML and MDS,^81^, ^82^ with demonstrated efficacy in combination with azacitidine including in patients with TP53‐mutant AML who are generally refractory to standard therapies. CD47 is also overexpressed in patients with MPNs and TP53 is a common driver of leukaemic transformation in patients with MPNs, suggesting potential utility here too.^83^, ^84^

## Vaccines and adoptive cell therapy

To date, the development of vaccination and CAR‐T cell therapies in myeloid disease have focussed on AML and not extensively implemented in MPNs. Wilms tumour 1 (WT1) is frequently over‐expressed in AML, and has been targeted by several vaccine trials with modest success.^85^ ‘Personalised’ vaccine approaches have also been tested, e.g. where a patient’s leukaemic blasts are fused with autologous dendritic cells, generating a hybridoma that is then reinfused into the patient.^86^ There are several CAR‐T cell trials targeting putative AML antigens including C‐type lectin‐like molecule‐1 (CLL‐1), CD33 and CD123,^87^ which if successful could also have activity in MPN, especially in patients with accelerated phase disease or post‐MPN AML.

The safety and efficacy of vaccination with a peptide derived from the *CALR* exon 9 mutation was recently tested in a phase I clinical trial in 10 patients with mut*CALR* MPN.^88^ Although a decline in platelet count was observed in the initial 100‐day period after vaccination, unfortunately none of the patients had either a clinical or molecular response. In this study, IFNγ enzyme‐linked immunospot (ELISPOT) responses were observed in four of 10 patients before vaccination, and in four additional patients after vaccination, confirming an increase in *in vitro* anti‐cancer immune activity and a potential role for therapeutic vaccines combined with other modalities to enhance the efficacy of anti‐tumour T‐cell activity.^88^

## Immune checkpoint blockade

Increased expression of the immune checkpoint mediators programmed cell death‐protein 1 (PD‐1) and PD‐L1 dampens the ability of T cells to react to tumour neoantigens. Both *JAK2* ^V617F^ and *CALR* mutations enhance PD‐L1 expression.^36^, ^89^, ^90^ Increased expression of the immune checkpoint receptors PD‐1 and cytotoxic T‐lymphocyte protein 4 (CTLA‐4) on CD4^+^ and CD8^+^ T cells in MPN has been shown,^88^ and blockade of PD‐1 and CTLA‐4 can recover T‐cell reactivity against mutCALR *ex vivo*, and result in the production of IFNγ and TNFα.^87^ Early trials with pembrolizumab have shown a reduction in the number of PD‐1‐expressing cells and increased expansion of the CALR‐specific CD4^+^ and CD8^+^ T cells.^89^ Trials with other checkpoint inhibitors including nivolumab and ipilimumab are currently underway.

## Conclusions

The ‘holy grail’ in MPN therapy is a targeted approach able to selectively ablate mutant clone stem cells or provide a dramatic competitive advantage to wild‐type stem cells, enabling normal haematopoiesis to recover. There is a clear rationale for immunomodulatory therapy, strongly backed by decades of experience with agents such as steroids, IFN and JAK‐inhibitors. More comprehensive profiling of the immune cell and protein repertoire in MPNs will be an important step in facilitating design of effective immunotherapies, as well as providing better insights into mechanisms and extent of immunocompromise and vulnerability to infection. Future treatments that harness the immune system may be highly effective; however, a personalised approach may be necessary given the vast heterogeneity between patients.

## Author contributions

All three authors agreed the scope of the article, reviewed the relevant literature, wrote the manuscript and designed the figures.
